# Handling coarsened age information in the analysis of emergency department presentations

**DOI:** 10.1186/s12874-020-01181-x

**Published:** 2020-12-07

**Authors:** Rhonda J. Rosychuk, Jeff W.N. Bachman, Anqi Chen, X. Joan Hu

**Affiliations:** 1 3-524 Department of Pediatrics, University of Alberta, Edmonton Clinic Health Academy, Edmonton, T6G 1C9 Canada; 2 Department of Mathematical and Statistical Sciences, University of Alberta, Edmonton, Canada; 3 Department of Statistics and Actuarial Science, Simon Fraser University, Burnaby, Canada; 4 Edmonton, Canada

**Keywords:** Administrative health datasets, Coarsened data, Doubly censored data, Recurrent events

## Abstract

**Background:**

Administrative databases offer vast amounts of data that provide opportunities for cost-effective insights. They simultaneously pose significant challenges to statistical analysis such as the redaction of data because of privacy policies and the provision of data that may not be at the level of detail required. For example, ages in years rather than birthdates available at event dates can pose challenges to the analysis of recurrent event data.

**Methods:**

Hu and Rosychuk provided a strategy for estimating age-varying effects in a marginal regression analysis of recurrent event times when birthdates are all missing. They analyzed emergency department (ED) visits made by children and youth and privacy rules prevented all birthdates to be released, and justified their approach via a simulation and asymptotic study. With recent changes in data access rules, we requested a new extract of data for April 2010 to March 2017 that includes patient birthdates. This allows us to compare the estimates using the Hu and Rosychuk (HR) approach for coarsened ages with estimates under the true, known ages to further examine their approach numerically. The performance of the HR approach under five scenarios is considered: uniform distribution for missing birthdates, uniform distribution for missing birthdates with supplementary data on age, empirical distribution for missing birthdates, smaller sample size, and an additional year of data.

**Results:**

Data from 33,299 subjects provided 58,166 ED visits. About 67% of subjects had one ED visit and less than 9% of subjects made over three visits during the study period. Most visits (84.0%) were made by teenagers between 13 and 17 years old. The uniform distribution and the HR modeling approach capture the main trends over age of the estimates when compared to the known birthdates. Boys had higher ED visit frequencies than girls in the younger ages whereas girls had higher ED visit frequencies than boys for the older ages. Including additional age data based on age at end of fiscal year did not sufficiently narrow the widths of potential birthdate intervals to influence estimates. The empirical distribution of the known birthdates was close to a uniform distribution and therefore, use of the empirical distribution did not change the estimates provided by assuming a uniform distribution for the missing birthdates. The HR approach performed well for a smaller sample size, although estimates were less smooth when there were very few ED visits at some younger ages. When an additional year of data is added, the estimates become better at these younger ages.

**Conclusions:**

Overall the Hu and Rosychuk approach for coarsened ages performed well and captured the key features of the relationships between ED visit frequency and covariates.

## Background

Data coarsening gives rise to a variety of challenges in data analysis. In general, data may be missing for reasons such as limited study resources, incomplete reporting by researchers or study participants, non-mandatory fields in administrative databases, and privacy concerns. The nature of the coarsened data and the aims of the analysis often motivate solutions for addressing the problem. For example, double censoring, both left- and right-censoring (e.g., [1, 2]), naturally arises when the events are only observed within a range bounded by the entry and the end of a study, possibly together with the births of subjects or other limits. The censoring mechanism for doubly censored data is complicated and the censoring times are partially or completely missing for subjects ([3]). Doubly censored data are alternatively defined as doubly interval censored data. More analysis approaches for doubly interval censored data are presented in Chapter 8 of [4]. In general, coarsened observations on a variable, different from missing observations in the strict sense, still bear information on the variable. It is often desirable but challenging to make good use of the information in the data analysis.

This paper is concerned with a large, administrative, health data study where birthdate data for all patients were originally withheld for patient privacy reasons. Previously, we studied children and youth in Alberta who visited emergency departments (ED) for mental health reasons (April 2002 to March 2011). Hu and Rosychuk [3] developed procedures for estimating age-dependent effects on the intensity of recurrent events on the age timescale in the presence of coarsened ages due to missing birthdates and in the presence of double censoring and provided asymptotic results that showed the procedures are valid theoretically. Pietrosanu, Rosychuk, and Hu [5] subsequently examined how estimates differed under different distributions for missing birthdates and in situations with different sample sizes through empirical studies. These empirical studies could not be as comprehensive as desired because the true birthdates were unavailable.

With recent changes in the data access rules for Alberta data, we are able to request a new extract of the mental health ED data that includes patient birthdates for April 2010 to March 2017. The new extract provides an opportunity to empirically verify the Hu and Rosychuk (HR) approach for coarsened ages by comparing their estimates using the data with estimates under the true, known ages. This paper focuses on examining the performance of the HR approach under five scenarios: uniform distribution for missing birthdates, uniform distribution for missing birthdates with supplementary data on age, empirical distribution for missing birthdates, smaller sample size, and an additional year of data.

## Methods

### Study setting and data

Alberta is a Canadian province with over 4 million residents [6] and a uniform single-payer health system – the Alberta Health Care Insurance Plan (AHCIP) – that provides medically necessary health care. The Government of Alberta is the custodian of all administrative databases used in our study and Alberta Health Services (AHS) provided the data from two population-based data sources: the National Ambulatory Care Reporting System (NACRS) and the AHCIP. Each Alberta resident is given a lifetime unique personal health number that can be used to link individuals across datasets.

The NACRS [7] database contains data for individual-level ambulatory care in all 104 EDs in Alberta. The NACRS database records details on each ED visit, such as demographics of the patient (e.g., birthdate, age in years at ED visit, sex), visit timing (e.g., registration date/time, triage date/time, end of ED visit date/time), triage level, diagnoses, interventions, discharge disposition, and geographic location.

For this study, all ED visits during April 1, 2010, to March 31, 2017, were extracted for Alberta residents aged <18 years at the time of the visit and who presented for mental health reasons. ED visits were considered mental health visits if the primary diagnosis field on the NACRS record contained International Statistical Classification of Diseases and Related Health Problems, Tenth Revision, Canada (ICD-10-CA) [8] corresponding to psychoactive substance abuse, schizophrenia/schizotypal/delusional disorders, mood disorders, neurotic/stress-related disorders, behavioural syndromes, behavioural/emotional disorders, disorders of adult personality, unspecified mental disorder, or toxic effects of non-medicinal substances. Additionally, ED visits were considered mental health related if they contained intentional self harm codes in any of the diagnosis fields. The full details of the codes used are provided elsewhere [9].

In addition, there was an AHCIP data extract from the Annual Cumulative Registry File (CRF) database that provided the most reliable data at fiscal year end for birthdate, sex, and geographic location. These fields were linked for patients in the ED data extract. Our study uses data on birthdate, age in years at ED visit, age in years at fiscal year end, sex, health zone of residence, and a rural/urban indicator based on the second character in the individual’s postal code. We refer to these data as the PMHC (pediatric mental health care) data. Sex, rural/urban and health zone variables are very stable over time and can be assumed to be time-independent [3]. If some patients changed their residence regions (rural/urban) or health zones during the study, we used the data at their first ED visit. Sex, rural/urban and health zone variables become our time-independent covariates used in our modeling that follows.

The University of Alberta Health Research Ethics Board approved this study and deemed that individual consent was not required.

### Statistical methods

The data extraction window for this study is [ *W*_*L*_,*W*_*R*_]=[April 1 2010, March 31 2017]. We set the recurrent events on the age *a* timescale, where *a*∈[0,18) years. The outcome of interest, *N*_*i*_(*a*), is the number of ED visits made from birth to age *a* for subject *i*, *i*=1,…,*n*. For subject *i*, $\mathcal {H}_{i}(a)$ is the history information prior to age *a* and *Z*_*i*_ is the covariate vector. For our data, the history would include the age at each prior ED visit and the covariate vector is comprised of sex, rural/urban indicator, and health zone of residence.

We first describe the methods when the model assumes the regression coefficients are constant over age and the birthdates are all available. We then apply the analogous algorithm called Procedure A from [3] as an asymptotic solution using a range of possible values for individual unavailable birthdates. Next, we provide the approach that allows regression coefficients to vary over age and assumes birthdates are unavailable.

#### Regression coefficients constant over age

The Anderson-Gill (AG) model is a commonly used Cox regression model for the counting process of recurrent events. Here, we consider the special case where the counting process is a Poisson process. Thus, the AG model assumes that the covariate effects *β* do not vary with age and the intensity is independent of all history information given the covariates. Then, the conditional intensity function of the AG model is $\lambda (a|\mathcal {H}_{i}(a), Z_{i}) = \lambda _{0}(a) \exp \left (\beta 'Z_{i}\right),$ where *λ*_0_(*a*) is the unspecified common baseline intensity for all subjects.

When the birthdates, **B**={*B*_1_,*B*_2_,…,*B*_*n*_}, are available, we can solve the equation

1 $$ \begin{aligned} U_{n}(\beta|\mathbf{B})=\frac{1}{n}\int_{0}^{18}Y(u|B_{i})\left\{Z_{i}-\frac{{\sum\nolimits}_{l=1}^{n}Y(u|B_{l})Z_{l}e^{\beta Z_{l}}}{{\sum\nolimits}_{l=1}^{n}Y(u|B_{l})e^{\beta Z_{l}}}\right\}dN_{i}(u)=0 \end{aligned}  $$

for *β* to obtain the estimator $\tilde {\beta }^{AG}_{n}$, where *Y*(*u*|*B*_*i*_)=*I*(max(0,*W*_*L*_−*B*_*i*_)<*u*≤ min(18,*W*_*R*_−*B*_*i*_)).

When birthdates **B=**{*B*_1_,*B*_2_,…,*B*_*n*_} are all missing, Procedure A from [3] provides an asymptotic solution by using the range *I*_*i*_ of possible values of birthdate *B*_*i*_ that is determined based on the available data for the subject. For example, if a subject had an ED visit on January 1, 2011, at the age of 1, then the possible values for the birthdate are *I*=[January 1 2010, January 2, 2009]. It assumes that the missing birthdate *B*_*i*_ is an independent variable following a uniform distribution, *G*_*i*_(·)=Unif(*I*_*i*_), within a year for each subject *i*. We can use a sample mean obtained from many collections of generated *B*_*i*_’s to approximate the required expectation in implementing the approach. That is, we randomly generate *W* independent collections of birthdates $\mathbf {B}^{(w)}= \left \{B^{(w)}_{i}| i=1,\dots,n\right \}$, where $w=1,\dots,W$ and $B^{(w)}_{i}\sim \text {Unif}(I_{i})$, for all subjects.

With the generation of birthdates, the estimating function in (1) becomes

$$\begin{aligned} \bar{U}^{AG}_{n,W}(\beta)&= \frac{1}{W}\sum\limits_{w=1}^{W}U_{n}\left(\beta|\mathbf{B}^{(w)}\right)\\ &=\frac{1}{nW}\sum\limits_{w=1}^{W} \sum\limits_{i=1}^{n}\int_{0}^{18}Y\left(u|B_{i}^{(w)}\right)\\&\quad\left\{Z_{i}-\frac{{\sum\nolimits}_{l=1}^{n}Y\left(u|B_{l}^{(w)}\right)Z_{l}e^{\beta Z_{l}}}{{\sum\nolimits}_{l=1}^{n}Y\left(u|B^{(w)}_{l}\right)e^{\beta Z_{l}}}\right\}dN_{i}(u), \end{aligned} $$

and yields an alternative estimator $\tilde {\beta }^{AG}_{nW}$, where $Y\left (u|B_{i}^{(w)}\right) =I\left (\max \left (0,W_{L}-B^{(w)}_{i}\right)< u \leq \min \left (18,W_{R}-B^{(w)}_{i}\right)\right)$.

Practically speaking, if the birthdates are known for subjects, then the lower and upper bounds of *I*_*i*_ are the same and equal to the true birthdate *B*_*i*_, for $i=1,\dots,n$. Thus, we can regard the known birthdates as a special case of “generating” **B** with a probability of 1 and *W*=1.

#### Regression coefficients vary over age

Assuming effects are constant over age may be too restrictive and the AG model can be too simplistic for a particular dataset. Hu and Rosychuk [3] estimate the multivariate covariate effects *β*(*a*) at age *a*, where *β*(*a*) may vary with *a*. Conditional on *Z*_*i*_, the expected rate function is *E*{*dN*_*i*_(*a*)|*Z*_*i*_}= exp(*β*(*a*)^′^*Z*_*i*_)*dΛ*_0_(*a*), where *Λ*_0_(*a*) is the cumulative baseline rate function.

The time-varying covariate effects *β*(*a*) are estimated by solving the estimating equation *E*_*n*_(*γ*;*a*)=0 for *γ* (Equation 9 in [3]), and use the first component vector of the estimator for *γ* to estimate *β*(*a*). Specifically,

$$\begin{aligned} E_{n}(\gamma; a)& =& \frac{1}{n} \int_{0}^{18} K_{h}(u-a) \sum\limits_{i=1}^{n} \left\{ Z_{i}^{*}(u,a)-\tilde{\bar{Z}}_{n}^{*}(\gamma;u,a)\right\}d\tilde{N}_{i}^{*}(u), \end{aligned} $$

where $\tilde {\bar {Z}}_{n}^{*}(\gamma ;u,a)=\tilde {S}_{n}^{(1)}(\gamma ;u,a)/\tilde {S}_{n}^{(0)}(\gamma ;u,a)$, with *A*^⊗*q*^=1,*A* for *q*=0,1 and $\phantom {\dot {i}\!}\tilde {S}_{n}^{(q)}(\gamma ;u,a) = {\sum \nolimits }_{i=1}^{n} \tilde {Y}_{i}(u)Z_{i}^{*}(u,a)^{\otimes q} \exp \left (\gamma ^{'}Z_{i}^{*}(u,a)\right)$. Also $\phantom {\dot {i}\!}Z_{i}^{*}(u,a)=\left (Z_{i}^{'},(u-a)Z_{i}^{'}\right), \tilde {Y}_{i}(u)=\int _{0}^{\infty } Y(u|b)dG_{i}(b)$, and $d\tilde {N}_{i}^{*}(u)=\int _{0}^{\infty }\left \{ Y(u|b)dN_{i}(u)\right \}dG_{i}(b)$, and *K*_*h*_(·)=*K*(·/*h*)/*h* is a kernel function to facilitate estimation.

With the *W* independent collections of randomly generated birthdates **B**^(*w*)^,*E*_*n*_(*γ*;*a*) can be approximated by ${\bar {U}_{n,W}(\gamma ;a)= \frac {1}{W}{\sum \nolimits }_{w=1}^{W}U_{n}\left (\gamma ;a|\mathbf {B}^{(w)}\right),}$ where $U_{n}\left (\gamma ;a|\mathbf {B}^{(w)}\right)=\frac {1}{n}\int _{0}^{18}K_{h}(u-a){\sum \nolimits }_{i=1}^{n} Y\left (u,B^{(w)}_{i}\right) \left \{Z^{*}_{i}(u,a)-\bar {Z}^{*}_{n}(\gamma ;u,a) \right \}dN_{i}(u)$ as defined in Equation 6 of [3]. Then, we can obtain the estimator $\tilde {\beta }_{nW}(\cdot)$ by solving $\bar {U}_{n,W}(\gamma ;a)=0$. The full development and details are in Hu and Rosychuk [3].

### Data scenarios

We investigate several scenarios that compare the estimates obtained when the birthdates are missing and when they are available.

### Scenario 1: Uniform distribution for missing birthdates

This scenario answers the question of whether or not the assumption of a uniform distribution for birthdates was appropriate. We first conducted analyses using the AG and the HR approaches and the complete PMHC data with the missing birthdates uniformly generated from individual’s birthdate intervals given by the ED visit dates. Then, we examined the performance of the models by comparing the estimates with missing birthdates to the ones obtained with the known birthdates.

### Scenario 2: Uniform distribution for missing birthdates and additional age data

This scenario answers the question of whether or not the incorporation of the available additional age information narrows the birthdate intervals sufficiently to alter the estimates. The CRF provides some additional information on age: the age in years at the end of the fiscal year (March 31) is provided for each subject and each fiscal year of the study. This additional age data potentially narrows the birthdate intervals used in Scenario 1 for the analysis with missing birthdates.

### Scenario 3: Distribution for missing birthdates based on true birthdates

**Fig. 1:**
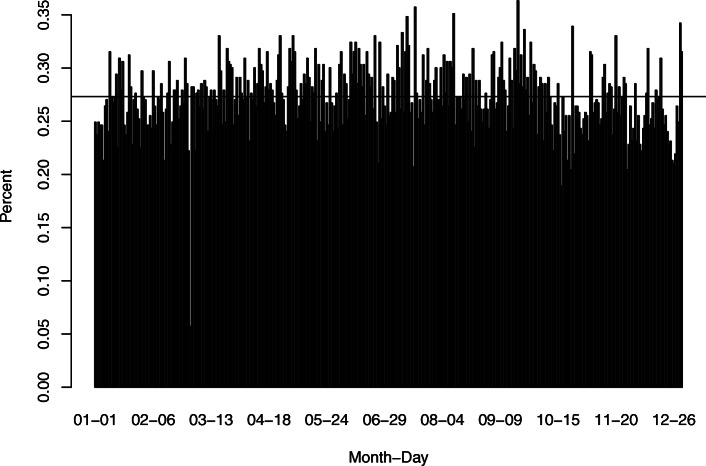
Empirical distribution of the birthdates

This scenario answers the question of whether or not another distribution for the missing birthdates would be better to use than the uniform distribution. The previous two scenarios assumed a uniform distribution for the true birthdate within a birthdate interval. The current data extract has the true birthdates and although these birthdates are not from the entire population of births in a single year, a proxy for the empirical distribution of birthdates can be created by ignoring the year and only focusing on the month and day of birthdates of subjects in the dataset (Fig. 1). With this approach, the probability of a birthdate within a birthdate interval would be relative to the empirical birthdate distribution with the interval. Figure 1 confirms that a uniform distribution of missing birthdates is a reasonable assumption.

### Scenario 4: Reduced sample size

The sample sizes for administrative data are often large and this scenario answers the question of the effect of missing birthdates on a smaller sample size. A random sample of 2,000 subjects and their 3,515 ED visits was taken from the dataset to explore estimates based on a smaller sample size.

### Scenario 5: Adding an additional year of data

This scenario answers the question of whether or not additional study time influences the performance of the estimates when birthdates are not available. An additional year of data would reduce the length of birthdate intervals for any subjects that had a visit in the additional time period. This scenario used the PMHC data from 2010 to 2016 and then compared results obtained with the full data to 2017.

For all scenarios, models with indicator variables for the three categorical covariates sex, rural/urban, and zone are fit (with female, urban, and the Edmonton zone as baselines) using specially developed C/C++ code that is called with R. We used *W*=5 and *W*=100 with the AG and HR models to investigate if the number of generated birthdates mattered when the birthdates were missing. Moreover, we used the Epanechnikov kernel and set two months as one time unit, the bandwidth to be 3 units, and the window of age to be [6, 102] time units. These are the same settings as in [3]. Since the differences between the local constant (LC) and the local linear (LL) estimates are very small [3], we only present the LC estimates in the following analyses.

## Results

### Patient characteristics

The PMHC data contain 58,166 ED visits made by 33,299 subjects. About 67% of subjects had one ED visit and less than 9% of subjects made over three visits during the study period. In addition, most visits (84.0%) were made by teenagers between 13 and 17 years old. Children under 6 years old had the least ED visits and made only 2.5% of visits. Among the 33,299 subjects, 57.7% were females, 78.4% lived in an urban area, and over a half resided in the largest metropolitan zones of Edmonton and Calgary (Edmonton: 25.1%; Calgary: 34.8%; North: 17.4%; Central: 14.3%; South: 8.4%).

### Scenario 1: Uniform distribution for missing birthdates

We started with the analysis under the AG model. Table 1 presents the estimates and the standard errors of the covariate effects for missing birthdates with *W*=5 and *W*=100 and the true birthdates. The differences among these estimates are tiny. Except for sex and South vs Edmonton zone, there is no significant effect of other covariates on the intensity of ED visits at a significance level of 5%.

**Table 1:** Coefficient estimates and standard error estimates under the Anderson-Gill model

	Missing birthdates					
Covariate	with *W*=5		with *W*=100		True birthdates	
	$\tilde {\beta }_{5}$	$SE\left (\tilde {\beta }_{5}\right)$	$\tilde {\beta }_{100}$	$SE\left (\tilde {\beta }_{100}\right)$	$\hat {\beta }(B)$	$SE\left (\hat {\beta }(B)\right)$
sex(male vs female)	-0.105	0.015	-0.104	0.015	-0.103	0.015
residence (rural vs urban)	-0.027	0.021	-0.027	0.021	-0.029	0.021
zone (South vs Edmonton)	-0.112	0.030	-0.113	0.030	-0.113	0.030
zone (Calgary vs Edmonton)	-0.034	0.021	-0.034	0.021	-0.035	0.021
zone (Central vs Edmonton)	-0.037	0.028	-0.037	0.028	-0.036	0.028
zone (North vs Edmonton)	-0.014	0.027	-0.014	0.027	-0.012	0.027

**Fig. 2:**
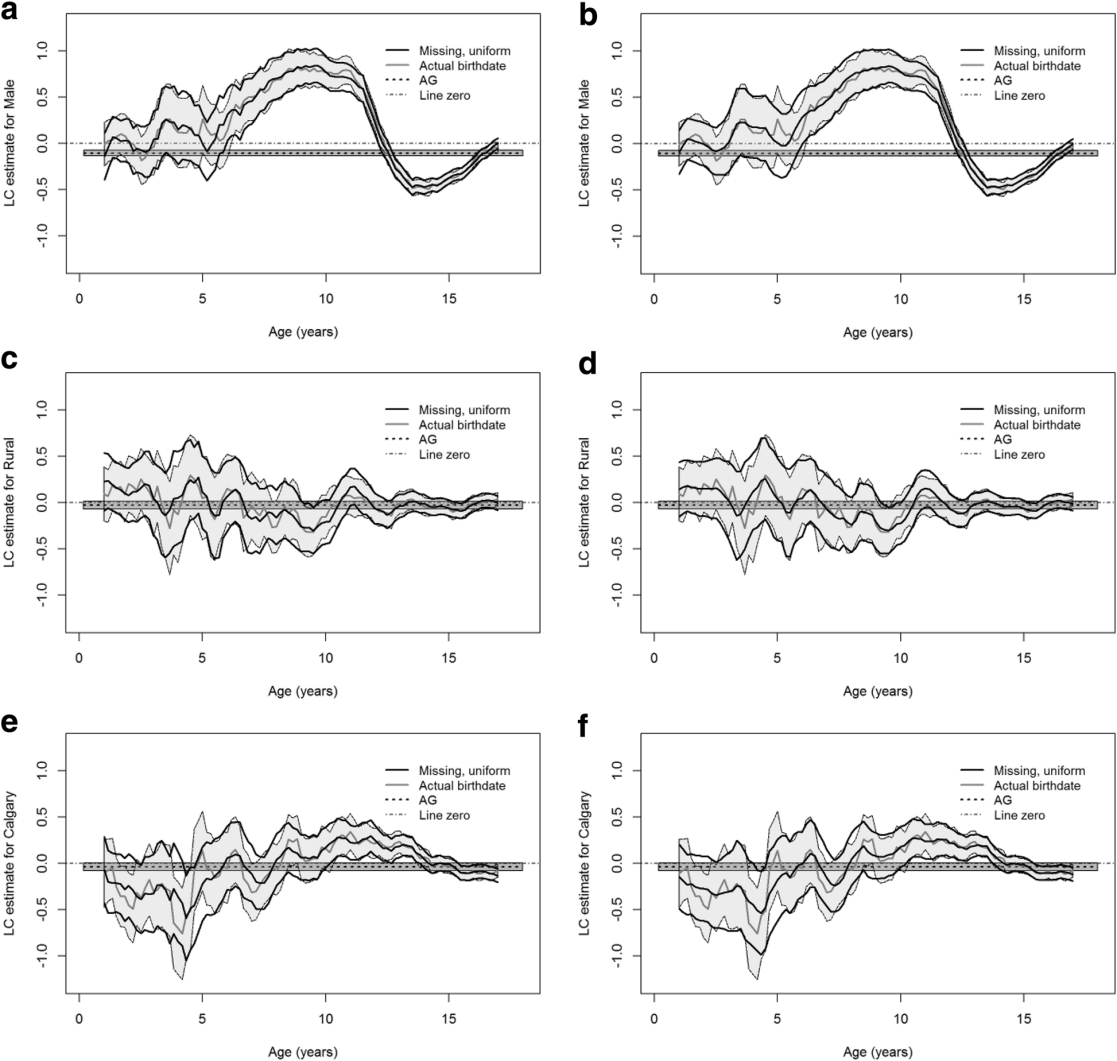
Local constant (LC) estimates and approximate 95% pointwise confidence intervals (CIs) for the effects of sex, rural vs urban residence and Calgary vs Edmonton zone using the full PMHC data. In each panel, the thick black curves denote the coefficient estimate and the corresponding 95% CIs under the Hu-Rosychuk (HR) model for missing birthdates with *W*=5 or *W*=100; the thick grey curve represents the estimate with the known birthdates and associated 95% CIs (shaded); the dashed line and the surrounding shade are the estimate and the 95% CIs under the Andersen-Gill (AG) model, respectively

Next, we conducted the analysis under the HR model. Figure 2 shows the estimates and the approximate 95% pointwise confidence intervals (CIs) of the covariate effects for missing birthdates with *W*=5 or *W*=100 and the true birthdates under the HR model together with those under the AG model. The estimate curves verify that the marginal regression model with the time-varying coefficients are more appropriate than the AG model with the time-independent coefficients. The estimates under the AG model can be regarded as the overall averages of the time-varying coefficient estimates [3]. The estimates with *W*=5 and *W*=100 both detect the main trends of the estimates for the known birthdates, suggesting the HR approach performs well regardless of the number of generated birthdates. The estimates with *W*=100 are the smoothest, as expected, and the ones for the known birthdates (i.e., *W*=1) are the least smooth.

There are some interesting findings indicated by the coefficient estimates of some covariates. In Fig. 2a and b, there is no significant difference on the frequency of ED visits between pre-school boys and girls. Boys at early school ages have significantly higher frequency of ED visits than girls at the same ages. However, the frequency of ED visits for teenage girls is significantly higher than that for teenage boys. Figure 2c and d suggest that there is no significant effect of rural/urban on the intensity of ED visits. In Fig. 2e and f, teenagers between 11 and 14 years old in the Calgary zone have significantly higher frequency of ED visits than teenagers at the same ages in the Edmonton zone; however, the frequency in the Calgary zone is not significantly different from that in the Edmonton zone at other ages. The graphs for the other zones do not differ substantially from the Edmonton zone (not shown).

### Scenario 2: Uniform distribution for missing birthdates and additional age data

About 25% of birthdate intervals have a width <331 days before the additional age data are used, whereas >75% have a width <331 days after the additional age data are used.

The additional age data result in only tiny changes for a few of the estimates in Table 1 (not shown). Since the coefficients are time-independent under the AG model, the whole dataset can contribute to the estimation of the coefficients and there would have to be substantive narrowing of many of the birthdate intervals to make an overall impact on the estimated effects.

**Fig. 3:**
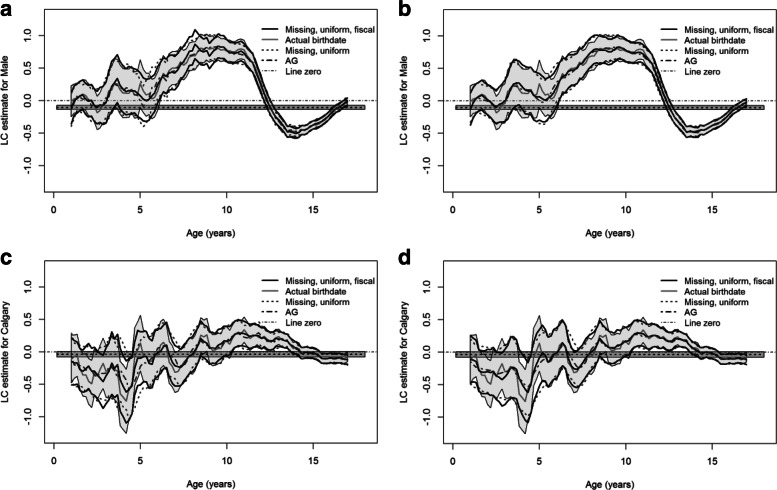
Local constant (LC) estimates and approximate 95% pointwise confidence intervals (CIs) for the effects of sex and Calgary vs Edmonton zone using the full PMHC data together with the fiscal year end data. In each panel, the thick black curves denote the coefficient estimate and the corresponding 95% CIs under the Hu-Rosychuk (HR) model for missing birthdates with *W*=5 or *W*=100; the thick grey curve represents the estimate with the known birthdates and associated 95% CIs (shaded); the dark grey dashed curves denote the estimate and the 95% CIs without the fiscal year end data; the dashed line and the surrounding shade are the estimate and the 95% CIs under the Andersen-Gill (AG) model, respectively

Figure 3 presents the LC estimates and the approximate 95% pointwise CIs for the effects of sex and zone using the full PMHC data and the fiscal year end data. The graphs for the rural areas do not differ significantly from the urban areas (not shown). Similar to the results for Scenario 1, the estimate curves with both *W*=5 and *W*=100 capture the main trends of the estimates using the true birthdates. Additionally, Fig. 3 indicates that including the additional age data does not result in a notable change in the coefficient estimates because adding the additional information does not lead to substantive narrowing of enough of the birthdate intervals to have an influence on the estimation.

### Scenario 3: Distribution for missing birthdates based on true birthdates

**Fig. 4:**
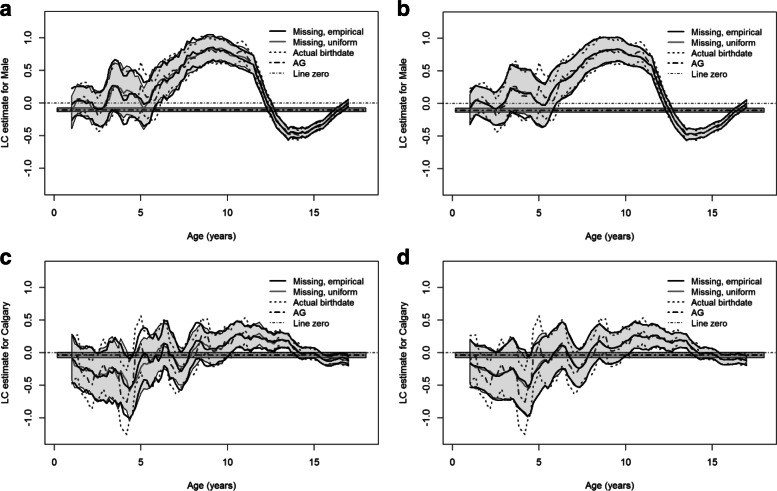
Local constant (LC) estimates and approximate 95% pointwise confidence intervals (CIs) for the effects of sex and Calgary vs Edmonton zone using the full PMHC data and the empirical birthdate distribution for missing birthdates. In each panel, the thick black curves denote the coefficient estimate and the corresponding 95% CIs for missing birthdates under the empirical distribution with *W*=5 or *W*=100; the thick grey curve represents the estimate for missing birthdates under a uniform distribution and associated 95% CIs (shaded); the dark grey dashed curves denote the estimate and the 95% CIs with the known birthdates; the dashed line and the surrounding shade are the estimate and the 95% CIs under the Andersen-Gill (AG) model, respectively

Figure 4 shows the coefficient estimates of sex and zone and the approximate 95% pointwise CIs with the full PMHC data and missing birthdates under the empirical birthdate distribution (Fig. 1). Figure 4 indicates that the estimates and the 95% CIs are almost identical for missing birthdates under a uniform distribution and the empirical distribution, particularly when *W*=100, because the empirical birthdate distribution is very close to a uniform distribution. Compared with the estimates for the known birthdates, the use of the empirical distribution does not clearly improve the estimation of the coefficients.

### Scenario 4: Reduced sample size

This scenario had a random sample of 2,000 subjects. More than a half (67.1%) of subjects had one ED visit and only 8.6% made over 3 visits. Most visits (82.5%) were made by teenagers whose ages are between 13 and 17 years old. Pre-school children made less than 3% of the visits. Among the 2,000 subjects, 56.7% were girls, 78.6% lived in an urban area, and more than a half (59.3%) resided in the Edmonton and Calgary zones.

**Fig. 5:**
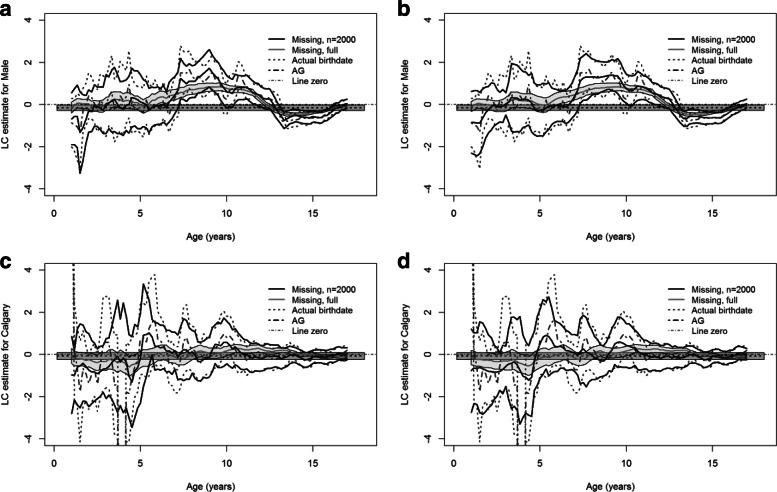
Local constant (LC) estimates and approximate 95% pointwise confidence intervals (CIs) for the effects of sex and Calgary vs Edmonton zone using a random sample of 2,000 subjects and their ED visits from the PMHC data. In each panel, the thick black curves denote the coefficient estimate and the corresponding 95% CIs using the reduced sample for missing birthdates with *W*=5 or *W*=100; the thick grey curve represents the estimate using the full data and associated 95% CIs (shaded); the dark grey dashed curves denote the estimate and the 95% CIs with the reduced sample and the known birthdates; the dashed line and the surrounding shade are the estimate and the 95% CIs under the Andersen-Gill (AG) model, respectively

Figure 5 presents the coefficient estimates of sex and zone and the corresponding 95% pointwise CIs for the small sample of the PMHC data. The main trends in the estimates of sex agree with the ones using the full PMHC data while the bands are much wider than the ones using the full data. Figure 5c and d present no significant effect of the Calgary zone on the intensity of ED visits; there are two big jumps at younger ages (cropped) in the estimate for the known birthdates because there were only a few ED visits at pre-school ages in the reduced sample.

### Scenario 5: Adding an additional year of data

This scenario used the PMHC data from 2010 to 2016 and then compared results obtained with the full data to 2017. This subset of the PMHC data consists of 48,827 ED visits made by 28,497 subjects. Most (67.9%) subjects had one ED visit and only 8.1% of subjects made over 3 visits. About 84.2% of the visits were made by teenagers between 13 and 17 years old. Children under 6 years old made less than 3% of the visits. Furthermore, 57.5% of subjects were females, 78.3% lived in an urban area, and more than a half (59.8%) resided in the Edmonton and Calgary zones.

**Fig. 6:**
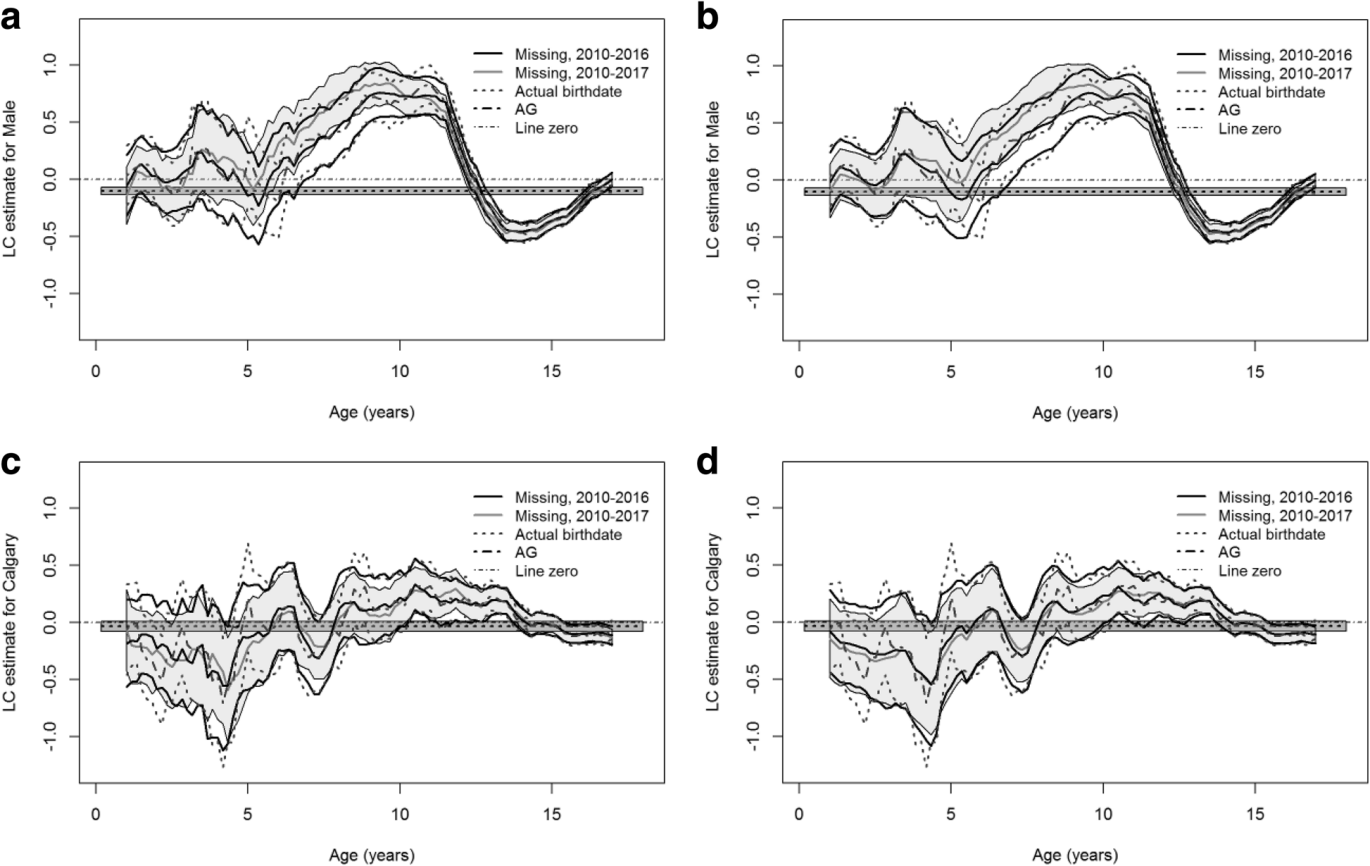
Local constant (LC) estimates and approximate 95% pointwise confidence intervals (CIs) for the effects of sex and Calgary vs Edmonton zone using the PMHC data from 2010 to 2016 and the full data. In each panel, the thick black curves denote the coefficient estimate and the corresponding 95% CIs using the data from 2010 to 2016 with *W*=5 or *W*=100; the thick grey curve represents the estimate using the full data and associated 95% CIs (shaded); the dark grey dashed curves denote the estimate and the 95% CIs with the known birthdates using the data from 2010 to 2016; the dashed line and the surrounding shade are the estimate and the 95% CIs under the Andersen-Gill (AG) model using the data from 2010 to 2016, respectively

Figure 6 presents the coefficient estimates of sex and zone with the approximate 95% pointwise CIs using the PMHC data from 2010 to 2016. The estimates detect the main trends of the estimates given by the true birthdates. In Figure 6a and b, the difference between the estimates using the six-year data and the full data is notable at ages between 5 and 10 and the estimates using the six-year data tend to be lower than the ones using the full data.

## Discussion

In this paper, we used a large, administrative PMHC data and the HR approach to estimate the age-dependent effects of sex, rural/urban, and health zone of residence on the intensity of ED visits for mental health reasons. The HR approach deals with coarsened censoring times due to the unreleased birthdates by randomly generating collections of possible birthdates from uniform distributions associated with study subjects. The availability of the true birthdates allows us to evaluate the performance of the HR approach against the known birthdates in different situations including the situation where there is useful additional age information, the situation where an empirical birthdate distribution for missing birthdates is applied, and situations where the sample size is smaller and additional years of data may become available. We found that the HR approach generally captures the main trends of the estimates given by the known birthdates for a large sample size regardless of the number of generated birthdates, *W*. As expected, the coefficient estimates are smoother for higher values of *W*.

Our results provide some practical insights on the application of the HR approach with the PMHC data. In Scenarios 2 and 3, we examined how additional age data and the use of the empirical birthdate distribution for missing birthdates influence the time-dependent coefficients for a large sample size, respectively. The results show that there is no meaningful change in the estimates in both scenarios. For simplicity, we can assume a uniform distribution for the missing birthdates and use the PMHC data excluding the fiscal year end data to estimate the coefficients in a large sample. In Scenario 4, we investigated the performance of the HR approach against the known birthdates and the full dataset for a small sample size. The estimates for missing birthdates detect the main trends of the estimates with the full data and the known birthdates for ages when data are available. There were some extreme estimates when there were few visits at lower ages and these jumps suggest that we would have to have a larger sample size to fully capture the effect across the age range. In Scenario 5, we were motivated by the ongoing study and evaluated how adding an additional year of data influences the estimates. The estimates have similar trends to the ones using the full dataset while the difference between the estimates for sex is notable for ages between 5 and 10. Therefore, including an additional year of data helps to increase the sample size and narrow birthdate intervals that may be especially important for ages where fewer ED visits occur and these changes improve the performance of the HR approach.

## Conclusion

Overall, the HR approach for missing birthdates with additional information from the database, which results in coarsened censoring times in the data analysis, performs well when compared to estimation using the known birthdates. Our paper provides insights on the practical aspects of applying this approach. Further, our examinations illustrate the effects of sample size on estimates, particularly for values of the time-varying covariate that may be less prevalent in the distribution of events.

## Data Availability

Data is the property of Alberta Health and the authors are not allowed to provide the data. Requests can be made for the same data from Alberta Health for researchers who meet the criteria for access to confidential data. Researchers are welcome to inquire for further information at Health.RESDATA@gov.ab.ca.

## Abbreviations

AG: Andersen and Gill
AHCIP: Alberta health care insurance plan
AHS: Alberta Health Services
CRF: Cumulative registry file
ED: Emergency department
HR: Hu and Rosychuk
ICD-10-CA: International Statistical Classification of Diseases and Related Health Problems, Tenth Revision, Canada
LC: local constant
LL: Local linear
NACRS: National ambulatory care reporting system
PMHC: Pediatric mental health care
